# UNDERSTANDING DEMENTIA PREVALENCE AND HEALTH CARE USE PATTERNS IN RURAL NORTH CAROLINA

**DOI:** 10.1093/geroni/igz038.3303

**Published:** 2019-11-08

**Authors:** Amanda N Grant, Tsai-Ling Liu, Nigel L Rozario, Deanna A Mangieri, Jennifer M Woodward, Yhenneko J Taylor

**Affiliations:** 1 University of North Carolina at Charlotte, Charlotte, North Carolina, United States; 2 Atrium Health, Charlotte, North Carolina, United States

## Abstract

Rural and remote communities have limited access to high quality dementia care, prompting a need for innovative solutions to meet the health care needs of affected older adults. As part of a study aimed at implementing a telehealth intervention for primary care patients with dementia in two rural North Carolina counties, we examined baseline dementia prevalence and compared health care use between patients with and without dementia. Electronic health records from January 2018 to December 2018 were examined for 2,288 patients aged 65 or older. A zero-inflated Poisson regression model was used to compare healthcare use between patients with and without dementia adjusting for patients’ demographic and clinical characteristics. Dementia prevalence was 8.7% based on diagnosis codes. Most patients with dementia were women (70%), not married (55%), Medicare-insured (78%), and had more comorbidities (mean: 2±2) than non-dementia patients. Dementia patients had a significantly higher number of primary care visits, emergency department visits, inpatient visits, and preventable hospitalizations than patients without dementia (risk ratio = 1.1, 1.8, 2.18, and 1.3, respectively; all P< 0.05). Dementia burden was higher among women and use of acute care services by patients with dementia in this rural setting was higher than patients without the disease, similar to urban settings. These findings suggest opportunities to improve care coordination and access to resources to help reduce the need for acute care services among patients with dementia and can help tailor interventions to address the health care needs of this group.

